# Thermoelectric Energy Conversion in a Lid-Driven Cavity Microgenerator Using Nanofluids

**DOI:** 10.3390/nano15181409

**Published:** 2025-09-12

**Authors:** Edgar Alexandro Gonzalez-Zamudio, Miguel Angel Olivares-Robles, Andres Alfonso Andrade-Vallejo

**Affiliations:** Instituto Politécnico Nacional, Sección de Estudios de Posgrado e Investigación, Escuela Superior de Ingenieria Mecanica y Electrica Unidad Culhuacan, Coyoacán, Mexico City 04430, Mexico; egonzalezz2400@alumno.ipn.mx (E.A.G.-Z.); aandradev@ipn.mx (A.A.A.-V.)

**Keywords:** lid driven, Richardson number, Organic TEG, SiO_2_ nanofluid

## Abstract

The present research seeks to characterize and evaluate a lid-driven cavity–TEG system to harness residual energy. Therefore, the behavior of water and a nanofluid ($SiO_{2}$) in a rectangular lid-driven cavity is numerically studied. The Navier–Stokes and energy conservation equations are solved using the finite difference method in Python. The fluid behavior is analyzed with a Reynolds number of 100, Richardson number of 100-77 and variable lid direction. Likewise, a thermoelectric module is integrated in the cavity, and the power generated by varying the size and number of thermocouples is studied. The results obtained contribute to the characterization of applicable thermal systems for their optimization. In the cavity, when the lid direction is positive, its interaction with the buoyant flow generates a vortex on the right side, and multiple vortices when it is in the negative direction; the isotherms present horizontal and vertical stratification in both cases. μTEG generates the most power with a 0.07 mm thermocouple size in the negative lid direction case, with an inlet gradient temperature of 8 K. $SiO_{2}$ (Ri = 77) showed a 23% increase in power output compared to water (0.318 μW/cm^2^ and 0.461 μW/cm^2^, respectively). With a 30% higher inlet gradient temperature ($SiO_{2}$ at Ri = 100, ΔT = 10.4 K, 0.569 μW/cm^2^), it generated 79% more power output compared to water.

## 1. Introduction

Heat transfer and fluid dynamics analyses are topics of current interest due to their wide-ranging applications in various fields. Among the typical engineering devices where fluid dynamics are significant are lid-driven cavities where fluids are the main driver; these have specific applications in industries such as electronic cooling, floating glass, and lake studies [1]. The fluid behavior analysis in these engineering systems is performed using numerical techniques based on the Navier–Stokes equations, which are inherently nonlinear [2].

Recent research on lid-driven cavities has highlighted the complexity of addressing these configurations, due to the intricate interplay between lid-driven flow and buoyancy, which remains largely unknown [3]. In recent research, An-Yang and Hang [4] studied a hybrid nanofluid in a square cavity subjected to a differential temperature and different convection regimes. They found that nanofluids, due to their high heat conduction capacity, tend to generate vortices in the streamlines, with nanoparticles playing a key role in determining the velocity and temperature distribution.

In turn, the analysis of nanoparticle combinations, as well as the addition of heat sources to generate controlled flows in a cavity, has been a central topic in recent research due to its multiple applications in diverse fields, such as industry and medicine [5]. Likewise, Elharfi et al. [6] simulated a nanofluid composed of water and copper nanoparticles in a rectangular lid-driven cavity (aspect ratio defined as the quotient between its length and height, A = L/H = 8), considering heat transfer on the lateral sides. They found that while nanofluids offer benefits in mixed convection situations, their effects remain unclear, particularly in situations involving a movable lid.

Research such as that of Sommerfeld and Taborda [7] addresses the effect of nanoparticles on the behavior of fluids at different Reynolds numbers, finding that the shear effect can influence the behavior of the fluid, depending on the Reynolds number, size and shape of the nanoparticles. This impact is more relevant the larger the particles are, particularly in the order of micrometers. Meanwhile, studies such as that by Bernagozzi et al. [8] analyze battery thermal management based on looped heat pipes and graphite sheets, showing that the use of LHP contributes to reducing the average module temperature, with greater effectiveness at high ambient temperatures. Systems of this type could be further enhanced using metallic suspensions in the form of nanofluids, as they increase thermal conductivity and improve heat transfer.

Nanofluids are of particular interest for improving heat transfer in heat exchangers, which is why Ho et al. [9] investigated the numerical uncertainty that can arise when using $Al_{2}O_{3}$ nanofluids; they found that the equations used to represent effective viscosity and thermal conductivity have a significant influence on the heat transfer characteristics obtained with the nanofluid. Corcione [10] delved into nanofluids with $Al_{2}O_{3}$ or Cu nanoparticles; they showed how the volume fraction of the nanoparticles and their size affect physical parameters such as density, conductivity, and specific heat. Furthermore, they found a way to increase the volume fraction; the specific heat decreased linearly, and the density decreased conversely, while the effective conductivity increased logarithmically.

Recent work has shown water-based $SiO_{2}$ nanofluid to perform better than others; Khaleel et al. [11] analyzed different water-based nanofluids such as ${Al}_{2}O_{3}$, Cu, $TiO_{2}$, and $SiO_{2}$ in a lid-driven trapezoidal cavity under a mixed heat exchanger governed by a Richardson number ranging from 0.1 to 10 and a Reynolds number of 100, heated by the bottom wall and with a non-adiabatic top wall. Among the most relevant findings is that $SiO_{2}$ exhibits the best heat transfer properties, followed by $Al_{2}O_{3}$ and $TiO_{2}$, which exhibit similar behavior.

Zhou et al. [12] developed a numerical model of a system representing an artificial reef with a trapezoidal geometry, open at the top and subjected to pulsating water flows. This configuration is considered a lid-driven cavity, where the interaction of the lid and geometry generates complex flows. Panama et al. [13] emphasize the importance of studying the thermal distribution of water within rectangular aquariums, especially when a heat source is introduced. Understanding how heat is propagated in this controlled environment is essential to maintaining optimal conditions for fish. The study of lid-driven cavities not only has applications in aquatic biology but also in various fluid applications.

The previous literature review highlights the different applications of lid-driven cavities and their potential for optimization with nanofluids. Nevertheless, achieving higher thermal efficiency remains a relevant challenge. In this context, thermoelectric generating devices (TEGs) emerge as a promising alternative, since when coupled to this type of cavity, they can effectively harness the residual energy. TEGs contain two semiconductors, P-type (with excess holes) and N-type (with excess electrons), and can transform temperature differences into potential differences through the Seebeck effect. Likewise, thermoelectric devices must be analyzed in different scenarios to determine the power generated according to the temperature gradient to which they are subjected, regardless of the information provided by the manufacturer [14]. New TEG devices continue to emerge that have not yet been fully characterized, like μTEGs, which are especially useful in applications with small temperature gradients.

Beretta et al. [15] studied the behavior of μTEGs (micro thermoelectric generators) as wristbands under small temperature gradients, varying the thermocouple size as well as the fill factor, defined as ff = $A_{pn}/A_{pni}$, where $A_{pn}$ represents the area of the thermocouples and $A_{pni}$ is the area of the thermocouples and the insulator. Sabet et al. [16] investigated the integration of thermoelectric generator (TEG) modules into aeronautical systems to recover part of the excess heat. In their study, TEGs were placed on both the exhaust valve and the surface heat sinks used to remove the heat generated by the fuel system and power electronics. The results indicate that, when these modules are coupled to surface heat exchangers, a significant voltage is generated due to the contact between the hot air and the thermoelectric device in that area.

This study presents an analysis of the behavior of a fluid (water) and a nanofluid (water–$SiO_{2}$) subject to convection within a rectangular lid-driven cavity, with a temperature differential across the sidewalls. This is due to the use of this type of configuration in different areas. Python 2.5 is used for numerical analysis, using the finite difference method. A micro thermoelectric generator (μTEG) module is also integrated into the bottom of the cavity. Different thermocouple sizes and fill factors (ff) are considered to determine the configuration with the highest output power. Finally, the streamline distributions, isotherms, temperature profiles inside the cavity, and the power generated by the μTEGs are assessed. The contribution of this research lies firstly in the characterization of the thermal and dynamic behavior of $SiO_{2}$ nanofluids; secondly in characterizing μTEG devices fabricated with uncommon materials; and finally in evaluating and proposing a potential real-world application of nanofluids and μTEGs.

## 2. Materials and Methods

The purpose of this section is to present the numerical procedure adopted in this study. First, the characteristics of the cap-driven cavity under investigation are described. Then, the governing equations of the nanofluids and their thermophysical properties are introduced. The following subsection details the characteristics of the thermoelectric generator (μTEG) model employed. Next, the coupled cavity–TEG configuration is presented, along with the equations that characterize the overall system. Afterward, the numerical procedure used to solve the problem is explained. Finally, the validation of both the μTEG model and the cavity simulations is provided.

### 2.1. Lid-Driven Cavity

The present research considers a lid-driven rectangular cavity with an aspect ratio A = 4.17, defined as the ratio of its length to height (A = L/H). Movement of the top lid and a thermal gradient along the side walls is contemplated, with a high temperature $T_{h}$ on the left wall and a lower $T_{c}$ on the right, with $T_{h}$ > $T_{c}$. The working fluids are water and nanofluid (10% volume fraction of $SiO_{2}$ in water), with a Prandtl number ($Pr=\mu C_{p}/k)$ of 7 [6] and 6.22 [11,17,18], respectively. The decision to concentrate on $SiO_{2}$ arises from its established superiority in heat transfer properties as demonstrated in prior research [11]. The fluid is treated as Newtonian and incompressible, with density variations addressed through the Boussinesq approximation concerning thermal flotation. The interaction of the fluid with the walls was examined using the no-slip condition. Additionally, thermal viscous dissipation and the stagnation of nanoparticles were considered negligible due to the consistent movement of the lid.

### 2.2. Richardson Number, Reynolds Number and Nanofluid Properties

The Richardson number, which in the context of a lid-driven cavity reflects the ratio of buoyancy forces to inertial forces, can be calculated using Equation (1):

(1) $$Ri\;=\frac{g\beta \Delta TL}{u^{2}}$$

where *g* represents the gravitational acceleration, β denotes the thermal expansion coefficient, Δ*T* is the temperature difference, *L* is the characteristic length, and *u* is the characteristic velocity. The Reynolds number is expressed as Re =ρuL/μ, where ρ indicates the density and μ signifies the dynamic viscosity.

The values for the density, dynamic viscosity, heat capacity, and thermal conductivity of the nanofluid at different volume fractions in water are presented in Table 1 [11,17,18]. The physical properties of nanofluids were estimated using the characteristics of the base fluid and nanoparticles reported by Khaleel et al. [9] (Table 2), along with Equations (2)–(6):

Volumetric expansion coefficient [11]:

(2) $${\left(\rho \beta \right)}_{nf}\;=\;(1\;-\;\varphi ){\left(\rho \beta \right)}_{f}\;+\;\Phi {\left(\rho \beta \right)}_{np}$$

Thermal conductivity [11]:

(3) $$\frac{k_{nf}}{k_{f}}=\frac{k_{np}+2k_{f}-2\Phi (k_{f}-k_{np})}{k_{np}+2k_{f}+\Phi (k_{f}-k_{np})}$$

Dynamic viscosity [18]:

(4) $$\mu_{nf}=\frac{\mu_{f}}{{\left(1-\Phi \right)}^{1.5}}$$

Density [11]:

(5) $$\rho_{nf}=\left(1-\Phi \right)\rho_{f}+\Phi \rho_{nf}\;$$

Heat capacity [11]:

(6) $${\left(\rho C_{p}\right)}_{nf}=\left(1-\Phi \right){\left(\rho C_{p}\right)}_{f}+\Phi {\left(\rho C_{p}\right)}_{np}$$

### 2.3. Thermoelectric Microgenerator

The integration of a μTEG device into the lower wall of the cavity is contemplated. Organic μTEG devices, unlike their traditional counterparts, offer the advantage of enhanced flexibility, enabling integration into both rigid and flexible systems while also being suitable for operation under low temperature gradients. Following Beretta et al. [15], the present model is composed of poly(ethylene 2,6-naphthalate) (PEN) substrates of 30 mm thickness. The properties of the P (PEDOT:PSS) material are $\sigma =3.6\times 10^{3}\;\Omega^{-1}m^{-1}$, $\alpha =15\;\mu VK^{-1}$, and $k=0.15\;Wm^{-1}K^{-1}$, which correspond to the electrical conductivity, the Seebeck coefficient, and the thermal conductivity, respectively. For the N (PDI) material, the properties are $\sigma =50\times 10^{3}\;\Omega^{-1}m^{-1}$, $\alpha =-167\;\mu VK^{-1}$ and $k=0.2\;Wm^{-1}K^{-1}$.

### 2.4. Actual System

**Figure 1:**
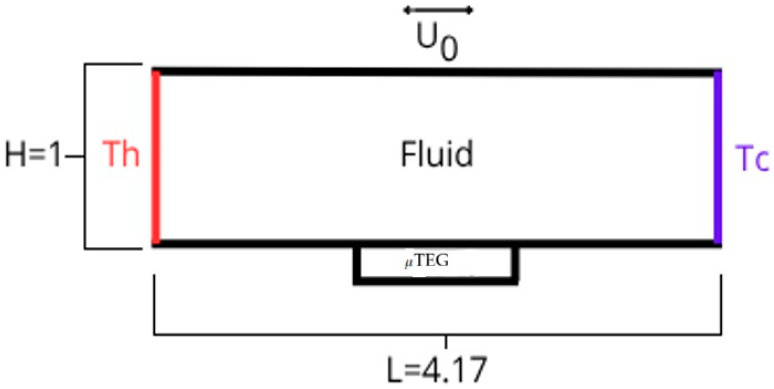
Dimensionless cavity with aspect ratio of 4.17.

Figure 1 presents the dimensionless operating conditions for analyzing the interaction between natural convection flow and forced flow, with a Reynolds number of 100 maintained in all fluids. Water will be maintained at a Richardson number of 100, while two cases will be considered in the nanofluid, one at Ri = 100 and another at Ri = 77, to observe its behavior by equalizing the dimensionless and dimensional conditions with water. Dimensionally, the cavity measures 4 cm (H) high and 16.7 cm (L) long and takes a minimum temperature of 300 K (which corresponds to the temperature at the cold wall $T_{c}$); it is subject to an 8 K gradient for water. In the first case of the nanofluid, the gradient will be 10.4 K (to be consistent with the Reynolds and Richardson numbers of 100). In the second case, a gradient of 8 K is contemplated for the nanofluid (equalizing the boundary conditions to those of water and having a Ri = 77). As mentioned, an integrated μTEG module is contemplated in the central part of the lower wall of the cavity, from L = 1 to L = 3.1, with 300 K as the cold temperature (lower side) and the temperature generated by the heat source from the left wall to the lower wall as the hot temperature (upper side). Then, the dimensionless boundary conditions of the cavity are (Equations (7)–(10))

(7) Left wall: U=V=0, θ=1

(8)  Right wall: U=V=θ=0

(9) $$Top\;wall:\;U=U_{0},\;V=0,\;\frac{d\theta }{dX}=0$$

(10) $$Bottom\;wall:\;U=0,\;V=0,\;\frac{d\theta }{dX}=0$$

### 2.5. Fundamental Equations

To perform the fluid simulation, the finite difference numerical method is employed within the Python 2.5 (Anaconda) programming environment. The equations solved in a dimensionless way are the mass conservation equation, the Navier–Stokes equation on the X and Y axes, and finally the energy conservation equation. These are shown below [19]:

(11) $$\frac{\partial U}{\partial X}+\frac{\partial V}{\partial Y}=0\;$$

(12) $$\left(\frac{\partial U}{\partial \tau }\right)+U\left(\frac{\partial U}{\partial X}\right)+V\left(\frac{\partial V}{\partial Y}\right)=-\frac{1}{Re}\left(\frac{\partial P}{\partial X}\right)+\frac{1}{Re}\left(\frac{\partial^{2}U}{\partial X^{2}}+\frac{\partial^{2}U}{\partial Y^{2}}\right)$$

(13) $$\;\left(\frac{\partial V}{\partial \tau }\right)+U\left(\frac{\partial V}{\partial X}\right)+V\left(\frac{\partial V}{\partial Y}\right)=-\frac{1}{Re}\left(\frac{\partial P}{\partial Y}\right)+\frac{1}{Re}\left(\frac{\partial^{2}V}{\partial X^{2}}+\frac{\partial^{2}V}{\partial Y^{2}}\right)+Ri\;*\;\theta$$

(14) $$\;\left(\frac{\partial \theta }{\partial \tau }+U\frac{\partial \theta }{\partial X}+V\frac{\partial \theta }{\partial Y}\right)=\frac{1}{Re\;*\;Pr}\left(\frac{\partial^{2}\theta }{\partial X^{2}}+\frac{\partial^{2}\theta }{\partial Y^{2}}\right)$$

Dimensionless variables of Equations (11)–(14) are as follows: *X* is the dimensionless distance in the horizontal coordinate, *Y* is the dimensionless distance in the vertical coordinate, *U* is the dimensionless flow velocity in the horizontal coordinate, *V* is the dimensionless flow velocity in the vertical coordinate, τ is the dimensionless time, *P* is the dimensionless pressure, and θ is the dimensionless temperature. They are defined as X=x/H, Y=y/H, $U=u/u_{0}$, $V=v/u_{0}$, $\tau =tu_{0}/H$, $P=(H\;*\;p)/(\mu \;*\;u_{0})$ and $\theta =(T-T_{c})/(T_{h}-T_{c})$, where *x* is the dimensional distance in the horizontal coordinate, *y* is the dimensional distance in the vertical coordinate, *H* is the height of the cavity, *u* is the flow velocity in the horizontal coordinate, *v* is the flow velocity in the vertical coordinate, $u_{0}$ is the maximum velocity in the horizontal coordinate, *t* is the time, *p* is the pressure, *T* is the temperature, $T_{h}$ is the maximum temperature, $T_{c}$ is the minimum temperature, and mu is the dynamic viscosity of the fluid. Regarding the dimensionless variables, *Re* is the Reynolds number, *Pr* is the Prandtl number, and *Ri* is the Richardson number.

The numerical simulation of the μTEG device is performed using Python with the code generated by Beretta [20]. The numerical simulation of the μTEG thermoelectric power is based on the conservation of energy across nodes; that is, a heat flow is considered through a hot reservoir, a hot substrate, a heat inlet and outlet in the thermocouple, a cold substrate, and a cold reservoir. Consequently, the following are true:

(15) $${\dot{q}}_{h}=h_{r,h}(T_{r,h}-T_{s,h})\;$$

(16) $${\dot{q}}_{h}=h_{s,h}\left(T_{s,h}-T_{h}\right)$$

(17) $${\dot{q}}_{h}={\dot{q}}_{\pi ,h}-\frac{\dot{q_{J}}}{2}+h_{pni}\left(T_{h}-T_{c}\right)$$

(18) $${\dot{q}}_{c}={\dot{q}}_{\pi ,h}+\frac{\dot{q_{J}}}{2}+h_{pni}\left(T_{h}-T_{c}\right)$$

(19) $${\dot{q}}_{c}=h_{s,h}\left(T_{c}-T_{s,c}\right)\;$$

(20) $${\dot{q}}_{c}=h_{r,c}\left(T_{s,c}-T_{r,c}\right)\;$$

where $\dot{q},\;h,\;T,\;{\dot{q}}_{\pi }\;and\;{\dot{q}}_{J}$ are the heat flux per unit area, the overall heat transfer coefficient, the Peltier heat flux, and the Joule heat flux, respectively. The subscripts h, c, r, s, pni represent hot, cold, reservoir, substrate, and thermocouple legs along with the insulator. The power of the μTEG, on the interval of [$10^{-6}$] to [$10^{-2}$] m, is given by [15]

(21) $$p_{out}=\frac{FF\alpha_{pn}^{2}}{\rho_{pn}l}\frac{m}{{\left(1+m\right)}^{2}}{\left(T_{h}-T_{c}\right)}^{2}$$

where $p_{out},\;FF,\;\alpha_{pn},\;\rho_{pn},\;l\;and\;m$ are the output power, the filling factor, the Seebeck coefficient of the thermocouples, the electrical resistivity of the materials (PEDOT:PSS-PDI), the thermocouple length (variable), and the load resistance ratio ($R_{load}/nR_{pn})$. Electrical resistivity, as well as other parameters such as those proposed by Beretta et al., were considered.

### 2.6. Numerical Procedure

The fluid simulation was carried out in the Jupyter Notebook (Version 3.1) environment, on a computer with an 11th Gen Intel(R) Core i5-1135G7 processor. A 400 × 96 node mesh and a time step of $10^{-4}$ were selected. To solve fluid dynamics, the finite difference method was used, with the equations of mass conservation, momentum, and energy [19]. The pressure was calculated using the Poisson equation [21], with all equations being solved simultaneously during each time iteration. The number of iterations was determined through validation efforts, with velocity changes being minimal, specifically less than 10^−8^. Once the simulation was performed, the temperature of the lower wall was extracted and considered as the hot temperature in the thermoelectric generator.

**Figure 2:**
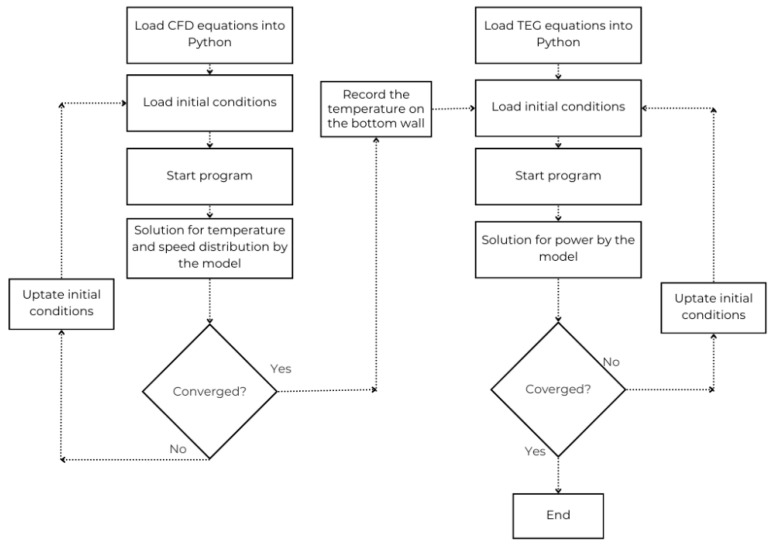
Numerical procedure flowchart.

The μTEG device was simulated in Python code, and the solution included six nonlinear equations that represent the energy balance across the device. The system was solved using Newton’s method, using a tolerance of 10^−8^ as a convergence criterion. Once the convergent solution was obtained, the output power was calculated from the temperature difference, Seebeck coefficients, and electrical resistance, using the classic relationship of the thermoelectric model, incorporating the load factor and the effective area [15]. The following (Figure 2) numerical procedure flowchart was considered:

### 2.7. Independence and Validation of Mesh

**Figure 3:**
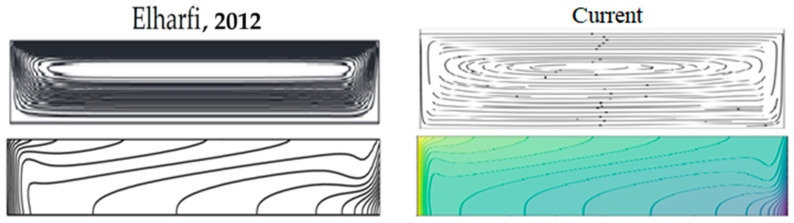
Streamlines and isotherms of $H_{2}O$, Ri = 10, Re = 10 Compared with Elharfi et al. [6] (2012).

**Figure 4:**
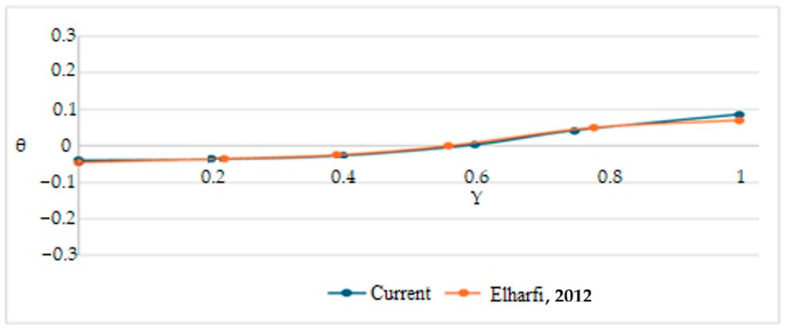
Dimensionless temperature at X = 4 of $H_{2}O$, Ri = 10, Re = 10 compared with Elharfi et al. [6].

The code validation of CFD was carried out by comparing it with the results reported by Elharfi et al. [6], who studied the behavior of a fluid (water with the addition of copper nanoparticles at different concentrations) in a rectangular cavity with an aspect ratio of A = 8. As shown in Figure 3, both the streamlines and the isotherms exhibit good agreement for the case of water with a Richardson number of Ri = 10. Likewise, Figure 4 shows a similarity in the dimensionless temperature profile along X = 4. Additional validations are in Appendix A.

**Figure 5:**
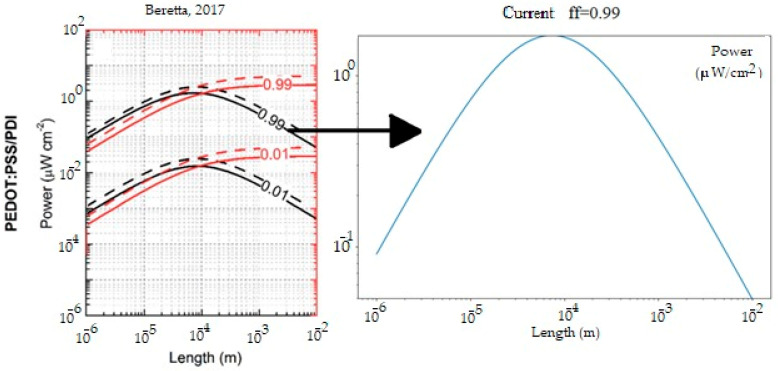
Power generated by μTEG PEDOT:PSS-PDI with temperature differential of 5 K compared with Beretta [15].

In the case of the μTEG, validation was performed using the work previously presented by [15], which simulated the output power of a PEDOT:PSS-PDI μTEG. In Figure 5, it can be observed how the graph with a filling factor (ff, ratio of conductive areas to the total surface of the module) of 0.99 in the μTEG shows exact agreement with what was previously reported, corresponding to the black line of 0.99. Additionally, Table 3 shows the numerical data.

## 3. Results

This section presents the results obtained. First, the outcomes of the mesh independence test and the calculation time are given. Then, the distributions of isotherms, streamlines, and temperature profiles, evaluated along the cavity midpoint and bottom wall, are presented for both positive and negative $U_{o}$. Subsequently, the power generated by the TEG device in each coupled system is analyzed. Finally, a comparison is made between the power generated using water and the nanofluid under different thermal gradients of the cavity with a positive lid direction.

### 3.1. Mesh Fluids

**Figure 6:**
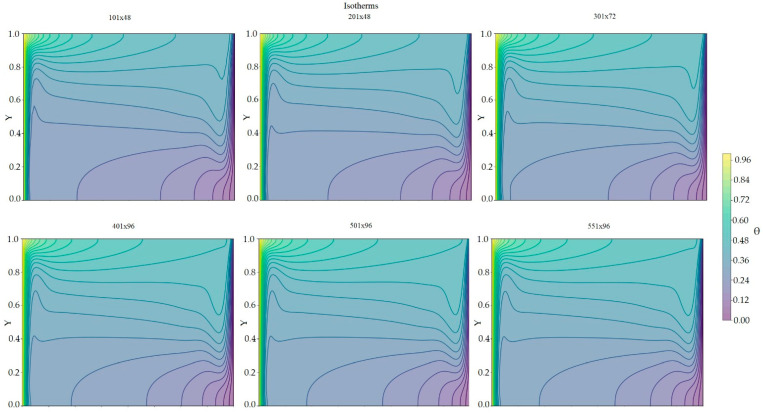
Isotherms of $SiO_{2}$ and $U_{0}$ at different mesh sizes.

Figure 6 shows various meshes and isotherms generated for $SiO_{2}$ with the cavity driven by the lid in the positive direction. Starting with a 301 × 72 mesh, the average dimensionless temperature and dimensionless velocity at half of the cavity (considering the temperature along Y) show no changes from the 401 × 96 mesh, presenting the same isotherms as the finer mesh. This can also be observed in Table 4, where, starting with a 401 × 96 mesh, the dimensionless temperature and velocity at the center of the cavity remain constant. In turn, the 401 × 96 mesh proves to be the most optimal in terms of computation time.

A comprehensive sensitivity test was carried out to analyze the effects of varying volume fractions of the nanofluid within a specific mesh framework. The mesh utilized for this study had dimensions of 401 × 96, allowing for a detailed examination of thermal behavior. The volume fraction of the nanofluid was systematically adjusted between 4%, 8%, and 10%, facilitating an in-depth exploration of its influence on thermal performance. The findings revealed that the mesh exhibited robust sensitivity to changes in the volume fraction, as evidenced by a noticeable increase in the dimensionless temperature measured at the midpoint along the height, denoted as X = L/2. The detailed results of these observations are compiled and presented in Table 5, offering a clear overview of the relationship between volume fraction and thermal response within the system.

### 3.2. Fluid Dynamics in Lid-Driven Cavity

**Figure 7:**
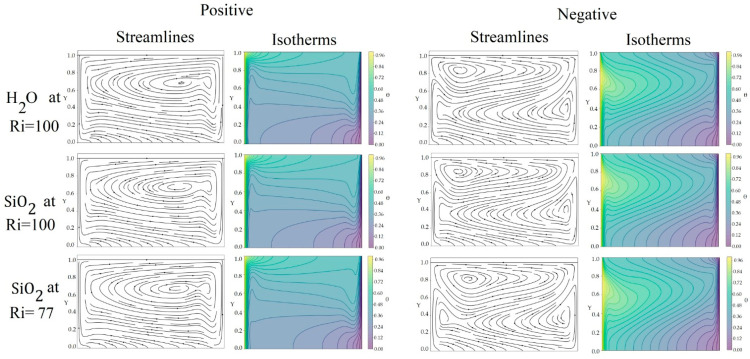
Streamlines and isotherms of water and $SiO_{2}$ at Re = 100.

Figure 7 shows the streamlines and isotherms for the different case studies, with the lid in the positive or negative direction. In cases where the lid is in the positive direction, there is a general tendency for the fluid to accumulate toward the cavity’s right wall. This phenomenon is a consequence of the strong interaction induced by the lid, which generates a shear flow that interacts with the flow due to the fluid’s buoyancy. This collision results in the formation of a dominant vortex on the right side of the cavity, favoring its accumulation in that region. When the lid is moving in the negative direction, in general, two vortices tend to be generated in the streamlines, one on the left side and one on the right side. This behavior occurs due to the interaction between the flow due to buoyancy and the lid movement, which generates a collision on the left side, in turn causing a vortex on the left side and another on the right side. The isotherms reveal a mixed stratification pattern, where the isotherms tend to elongate towards the right wall at approximately Y = 0.6; this is because the fluid displaced by the lid collides with the heated fluid, pushing it toward the right wall at this height.

**Figure 8:**
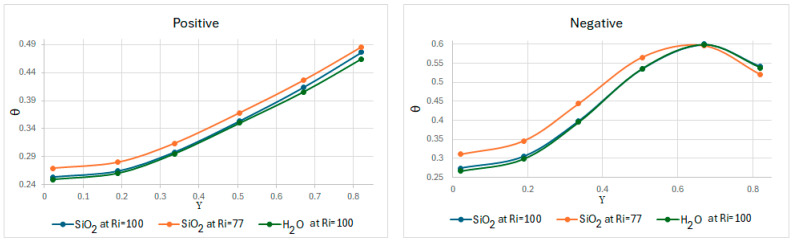
Dimensionless temperature along Y in X $=\frac{L}{2}$.

Figure 8 shows the dimensionless temperatures of the different fluids and cases along the *Y*-axis at X = L/2, with the lid in positive or negative directions. With the lid in the positive direction, an average dimensionless temperature of 0.343 for $SiO_{2}$ at Ri = 100, 0.357 for $SiO_{2}$ at Ri = 77, and 0.337 for water (Ri = 100) can be observed. Taking this average as a reference to describe the overall thermal behavior of the cavity, a temperature increase (from the initial temperature of 300 K) of approximately 3.59 K was observed for $SiO_{2}$ (Ri = 100), 2.85 K for $SiO_{2}$ (Ri = 77), and 2.70 K for water. It can be observed that the interaction of the lid helps to improve the temperature distribution along the cavity, increasing by 36% for $SiO_{2}$ (Ri = 77) and 34% $SiO_{2}$ (Ri = 100) and water. In the middle of the cavity, $SiO_{2}$ exhibited a higher dimensionless temperature at Ri = 77 than at Ri = 100. This behavior is because the second case exhibits stronger natural convection than the first case, which hinders heat transport to the middle of the cavity by reducing the interaction between forced and natural flows.

In turn, with the lid in the negative direction, an average dimensionless temperature of 0.443 can be observed for $SiO_{2}$ at Ri = 100, 0.464 for $SiO_{2}$ at Ri = 77, and 0.439 for water. When using this average as a reference to describe the overall thermal behavior of the cavity, a temperature increase of approximately 4.63 K was observed for $SiO_{2}$ (Ri = 100), 3.71 K for $SiO_{2}$ (Ri = 77), and 3.51 K for water. It can be observed how the interaction of the lid contributes to the temperature distribution throughout the cavity, generally increasing by 46% for $SiO_{2}$ (Ri = 77) and 44% for $SiO_{2}$ (Ri = 100) and water. In different cases, compared to when the lid has a positive direction, when the lid is in a negative direction, an increase of at least 22% in dimensionless temperature is observed in the middle of the cavity.

**Figure 9:**
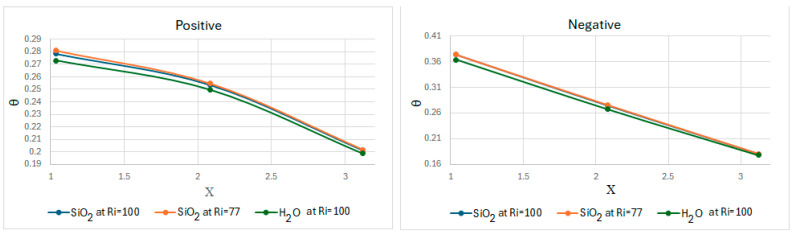
Dimensionless temperature at Y = 0 of water and $SiO_{2}$, under different lid directions on the bottom wall.

Figure 9 shows the dimensionless temperature profiles along the bottom wall of the cavity, both in the positive and negative directions, for the different cases. In general, for the different cases, it can be observed that the dimensionless temperature decreases progressively in the *X*-axis direction: with $SiO_{2}$ (Ri = 100), it is 28% less at X = 1 than X = 3, 27% less with $SiO_{2}$ (Ri = 77) and 27% less with water for the positive lid direction. In contrast, in the negative direction, the dimensionless temperature with $SiO_{2}$ (Ri = 100) is 52% less at X = 1 than X = 3, 59% less with $SiO_{2}$ (Ri = 77), and 51% less with water. The average dimensional temperature value on the bottom wall recorded with the lid in the positive direction is 301.92 K for water, 302.56 K for $SiO_{2}$ (Ri = 100), and 302.01 for $SiO_{2}$ (Ri = 77). With the lid in the negative direction, the dimensional temperature is 302.16 K for water, 302.89 K for $SiO_{2}$ (Ri = 100), and 302.52 K for $SiO_{2}$ (Ri = 77) at the bottom of the cavity. This is the temperature considered on the higher-temperature side of the thermoelectric module. This information is summarized in Table 6.

### 3.3. Generated Power: Effect of Fill Factor (Thermocouple Size)

**Figure 10:**
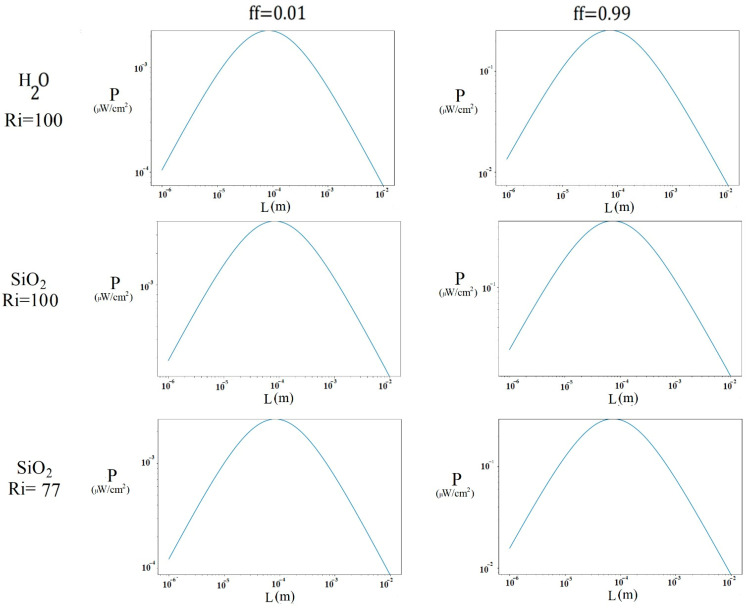
Power generated at different thermocouple lengths with the cavity lid in the positive direction.

Figure 10 shows the power generated by the thermoelectric module coupled to the cavity with the lid in the positive direction, considering a temperature differential (with a minimum temperature of 300 K) of 1.92 K for water, 2.56 K for $SiO_{2}$ (Ri = 100), and 2.010 K for $SiO_{2}$ (Ri = 77). In the different cases for ff = 0.01, it is observed that the maximum power output was obtained at a thermocouple size of 0.08 mm, being approximately 2.240 ${\times \;10}^{-3}$ μW/cm^2^ in the case of water, 4.04 ${\times \;10}^{-3}$ μW/cm^2^ in the case of $SiO_{2}$ (Ri = 100), and 2.630 ${\times \;10}^{-3}$ μW/cm^2^ in the case of $SiO_{2}$ (Ri = 77). With ff = 0.99, the maximum power was also obtained at a thermocouple size of approximately 0.07 mm, being 0.253 μW/cm^2^ in the case of water, 0.456 μW/cm^2^ in the case of $SiO_{2}$ (Ri = 100), and 0.297 μW/cm^2^ in the case of $SiO_{2}$ (Ri = 77). In the different cases, $SiO_{2}$ (Ri = 100) generated more power; however, its initial temperature in the cavity was 30% ([10.4 − 8]/8) higher than that of $SiO_{2}$ (Ri = 77), producing 53% ([0.456 − 0.297]/0.297) more power. Meanwhile, $SiO_{2}$ (Ri = 77) produced 17% ([0.297 − 0.253]/0.253) more power than water at the same inlet temperature.

**Figure 11:**
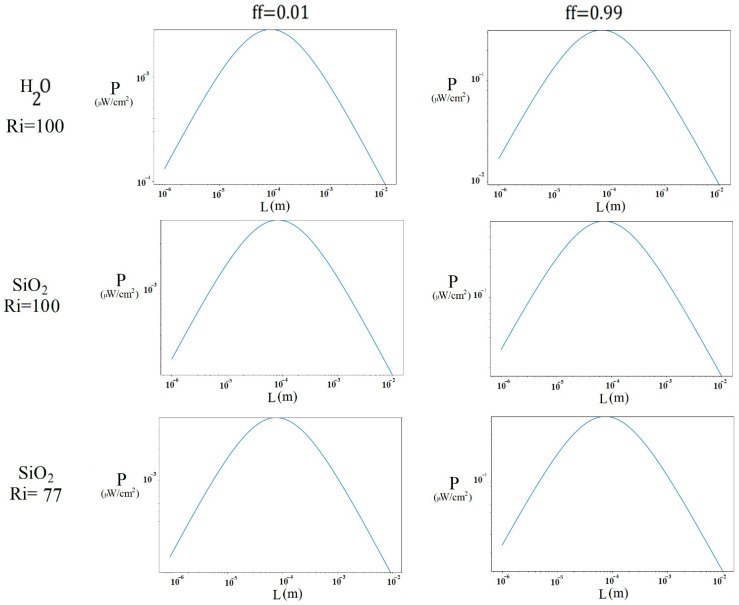
Power generated at different thermocouple lengths with the cavity lid in the negative direction.

Figure 11 shows the power generated by the thermoelectric module when considering a temperature differential of 2.16 K for water, 2.89 K for $SiO_{2}$ (Ri = 100), and 2.52 K for $SiO_{2}$ (Ri = 77), corresponding to the case where the direction of the lid in the enclosure is negative. In general, with ff = 0.01, the maximum power was obtained at a thermocouple size of approximately 0.08 mm, being 2.270 ${\times \;10}^{-3}$ μW/cm^2^ in the case of water, 5.047 ${\times 10}^{-3}$ μW/cm^2^ in the case of $SiO_{2}$ (Ri = 100), and 3.956 ${\times \;10}^{-3}$ μW/cm^2^ in the case of $SiO_{2}$ (Ri = 77). With an ff = 0.99, the highest power output was obtained at 0.07 mm, with 0.318 μW/cm^2^ for water, 0.569 μW/cm^2^ for $SiO_{2}$ (Ri = 100), and 0.461 μW/cm^2^ for $SiO_{2}$ (Ri = 77). Overall, the $SiO_{2}$ nanofluid (Ri = 100) produced approximately 23% ([0.569 − 0.461]/0.461) more power than $SiO_{2}$ (Ri = 77), requiring a 30% ([10.4 − 8]/8) higher initial temperature. On the other hand, $SiO_{2}$ (Ri = 77) achieved 45% ([0.461 − 0.318]/0.318) more power than water at the same cavity temperature. The difference in output power of $SiO_{2}$ at Ri = 100 and water was 79% ([0.569 − 0.318]/0.318).

Table 7 summarizes the power generated under the different cases. It can be denoted as the maximum output power obtained with $SiO_{2}$ at Ri = 100, with the cavity lid in the negative direction and an ff = 0.99 in the μTEG device. However, considering the inlet temperature in the cavity and the generated power, the most efficient case was $SiO_{2}$ (Ri = 77), an ff = 0.99, and the cavity in the negative lid direction.

### 3.4. Power: Effect of the Temperature Difference

**Figure 12:**
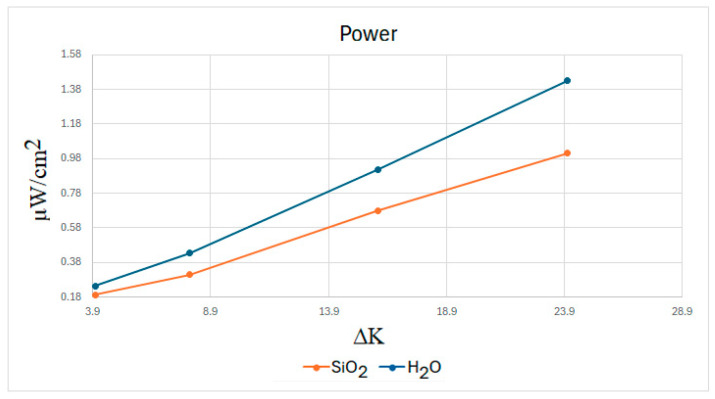
Power generated under different temperature gradients in the negative direction (ff = 0.99).

Based on the previous results, the power generated in the μTEG device in the lid-actuated cavity in the negative direction under different temperature gradients with $SiO_{2}$ and water was analyzed. It was analyzed with an ff = 0.99, and it was noted that the maximum power generated in the different cases was at a thermocouple size of approximately 0.07 mm, as observed in previous results. In Figure 12, it can be seen that as the temperature to which the fluids are subjected increases, the difference in power generated between the two fluids becomes greater, with $SiO_{2}$ being the best, as seen in Table 8.

## 4. Discussion

The heat transfer and dynamic behavior of a fluid contained in a rectangular cavity actuated by a lid were studied. Based on this analysis, the output power generated by a μTEG subjected to different thermocouple sizes was also evaluated. According to the study’s objective, it was identified that, based on the thermal gradient generated within the cavity, an optimal thermocouple size exists to maximize electrical power generation. The results showed that the thermocouple size significantly influences module efficiency, identifying that the specific value that allows the highest output to be achieved at ff = 0.01 was 0.08 mm, while at ff = 0.99, it was 0.07 mm. In turn, it was found that the lid in the negative direction contributed to improved heat transfer and consequent power generation more than the lid in the positive direction.

The behavior observed in Figure 7 can be explained as follows: when the lid is in the positive direction, the fluids present a central vortex due to the interaction of the lid and buoyancy flow in the streamlines, generating a clear pattern of horizontal stratification in the isotherms, which is more pronounced in the nanofluid with $SiO_{2}$ (Ri = 100) than in the water. This stratification is a direct product of the movement generated by the lid, which produces a more uniform thermal profile in the horizontal direction. The behavior with the negative lid direction, where two vortices tend to be generated in the streamlines, one on the left side and one on the right side, occurs due to the interaction between the flow due to buoyancy and the movement of the lid generating a collision on the left side, which therefore causes a vortex on the left side and another on the right side. In these cases, the isotherms reveal a mixed stratification pattern, where the isotherms tend to appear elongated towards the right wall at approximately Y = 0.6. This is because the fluid being pushed by the lid collides with the heated fluid, causing it to be pushed towards the right wall at this height. These behaviors are similar in both fluids.

Figure 9 and Figure 10 show that the $SiO_{2}$ nanofluid produced more power than water in the different cases. This is due to its superior heat transfer properties, which are best reflected when the same cavity inlet temperature is maintained for both water and $SiO_{2}$, resulting in a nanofluid that produces more power due to its improved thermal properties. In turn, the fact that the μTEG device achieves better generation at a higher fill factor is due to the increase in conductive areas ($A_{pn}$). The length of the thermocouple at which the greatest power is obtained may be due to the point where an effective temperature gradient is maintained. If the length is too short, heat is conducted quickly between the ends, reducing the gradient; on the other hand, if it is too long, heat conduction decreases excessively, limiting the useful heat flow.

Drawing from previous observations, it is evident that when the Richardson number exceeds 10 (Ri > 10), indicating a regime dominated by natural convection, the effects of buoyancy on fluid flow can either amplify or hinder the motion generated by the lid, contingent on its direction of movement. For instance, when the lid is displaced in the negative direction, the strong buoyancy forces engage dynamically with the flow induced by the lid, resulting in the upward movement of hot fluid towards the colder wall of the cavity. This interaction has profound implications, as it modifies the vortex structures within the fluid as well as the thermal stratification present in the cavity.

This intricate phenomenon becomes particularly highlighted in the comparison of thermal behaviors of $SiO_{2}$ at two different Richardson numbers, specifically Ri = 100 and Ri = 77: When the lid moves in a positive direction, the measured dimensionless temperature at the midpoint (X = L/2) is observed to be lower than that recorded when the lid moves in a negative direction. In turn, with the lid moving in a negative direction, the regime characterized by a lower Richardson number (Ri = 77) results in a higher dimensionless temperature, implying that the weaker buoyancy forces prevailing in this state allow the shear flow induced by the lid to more effectively facilitate thermal transport.

Thus, it becomes evident that the Richardson number plays a critical role in shaping fluid dynamics within the cavity and directly influences the performance metrics of the micro thermoelectric generator (μTEG), highlighting its significance in the design and optimization of thermal management systems.

The different thermal gradients presented by the nanofluid in the cavity affect the power generation; however, this power is also influenced by flow optimization, which shows better heat transfer with the lid oriented in the negative direction.

As shown in Figure 11, as the temperature gradient increases, the difference in power generated by the two fluids also increases, with the nanofluid exhibiting greater heat transfer than water. This behavior occurs because the greater the temperature gradient, the better the nanofluid reflects its thermal properties. In turn, as the temperature difference increases, the heat flux increases due to Fourier’s law, and consequently, so does the power generated in the μTEG.

**Figure 13:**
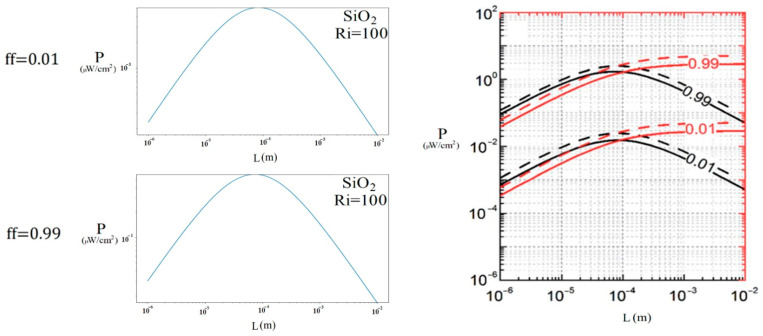
Power generated at different thermocouple lengths and currents, compared to previous studies [13].

Figure 13 compares the case where the μTEG generated the highest power (ΔT=2.888 K, $SiO_{2}$, Ri = 100, and negative lid direction) with previous studies [15] where the same type of μTEG (PEDOT-PSS:PDI) was analyzed under a 5 K gradient (solid black lines represent the generated power). In both cases, a similar behavior is observed: the power reaches its maximum value at a thermocouple length of 0.07 mm, exhibiting a parabolic trend at different sizes. Furthermore, it was found that the power is higher with a filling factor of 0.99 than at 0.01. Although the power generated in the present study was approximately 5 times lower, this is attributed to the fact that the temperature gradient was also about 2.5 times lower. The difference in power reflected is due to the difference in temperature, since the materials and parameters were the same. This suggests that the device’s behavior is not linear and that the generated power does not increase proportionally to the applied thermal gradient.

Therefore, when coupling thermoelectric generators to a lid-driven cavity, this work, compared to the results reported by other researchers, delves deeper into the potential uses of μTEGs, contributing to the characterization of these new technologies and the discovery of the best configurations to maximize energy efficiency. In general, the present findings open application possibilities in various industries, as well as in domestic environments. These include, for example, the use of waste heat in systems such as fish tanks, where energy could be recovered passively and continuously.

## 5. Conclusions

This comprehensive study provides a novel characterization of μTEG performance in lid-driven cavities with $SiO_{2}$ nanofluid, highlighting the nonlinear relationship between temperature gradients and power output in a previously unexplored system. To achieve this, we investigated the thermal and dynamic behavior of two fluids, water and a nanofluid composed of water and silicon dioxide ($SiO_{2}$), within a rectangular lid-driven cavity. The primary objective of the research was to assess the feasibility of these fluids for power generation via a micro thermoelectric generator (µTEG) and evaluate the applications to recover residual energy from lid-driven cavity systems.

The findings revealed that the motion direction of the cavity lid plays a critical role in determining the system’s efficiency. Notably, when the lid is oriented negatively, there is a marked improvement in heat transfer efficiency, which subsequently leads to enhanced power generation capabilities. This aspect is particularly significant when comparing the efficiencies of water and $SiO_{2}$ nanofluid at varying Richardson numbers (Ri). The data indicated that $SiO_{2}$ nanofluid exhibits optimal power generation at Ri = 100, while it produces the highest energy output at the lowest inlet temperature when the Richardson number is set to Ri = 77. These insights emphasize the importance of carefully choosing the Richardson number in the design and optimization of thermal systems that aim to harvest energy from the surrounding environment.

Greater power was generated when the TEG was provided with a higher temperature gradient, which was facilitated by optimizing the flow in the cavity through the lid direction. Maximum power generation was achieved when the lid moved in the negative direction. Furthermore, throughout the research, the optimal size of the thermocouple for maximizing power generation was determined to be 0.07 mm. This size demonstrated minimal variation in performance across different fluid types and fill factors (ff), suggesting a robust design parameter. A key finding is the nonlinear relationship between temperature gradient and μTEG performance, which points to a more complex, nonlinear behavior compared to other established studies.

Overall, the contribution of this research is to provide an in-depth analysis of the interactions between µTEGs and lid-driven cavities, thus paving the way for innovative applications in both industrial and residential contexts. By effectively harnessing waste heat sources, this approach has the potential to facilitate passive and sustainable energy generation, significantly contributing to energy efficiency and environmental sustainability efforts.

## Figures and Tables

**Table 1:** Physical properties of nanofluid $SiO_{2}$–water.

Volume Fraction (%)	$\rho \left(\frac{\mathrm{kg}}{cm^{3}}\right)$	$k\left(\frac{W}{m*K}\right)$	$C_{p}(\frac{J}{\mathrm{kgK}})$	$\mu (\;\mathrm{Pa}\times s\;{\times 10}^{-3})$	Pr
4	1046.080	0.631	3889.334	1.063	6.553
8	1094.160	0.649	3622.389	1.133	6.322
10	1118.20	0.659	3497.525	1.171	6.220

**Table 2:** Physical properties of $SiO_{2}$ and water.

	$\rho \left(\frac{kg}{cm^{3}}\right)$	$k\left(\frac{W}{m*K}\right)$	$C_{p}(\frac{J}{\mathrm{kgK}})$	$\mu \left(Pa\times s{\times 10}^{-3}\right)$
Water	998	0.613	4182.2	1.010
SiO_2_	2200	1.2	703	-

**Table 3:** Power generated by μTEG PEDOT:PSS-PDI with temperature differential of 5 K.

Length (m)	$\mathrm{Power}\;(\mu W/cm^{2})$	
	Beretta et al.	Current.
$10^{-3}$	${4\;\times \;10}^{-1}$	${4\;\times \;10}^{-1}$
$10^{-4}$	1.8	1.8
$10^{-5}$	${5\;\times \;10}^{-1}$	${5\;\times \;10}^{-1}$

**Table 4:** Computation time and dimensionless temperature.

Mesh	Time	Dimensionless Temperature at X = L/2	Dimensionless Velocity in X Direction at X = L/2
101 × 48	5 min and 58 s	0.298	0.079
201 × 48	8 min and 30 s	0.304	0.080
301 × 72	16 min and 7 s	0.330	0.078
401 × 96	29 min and 16 s	0.343	0.080
500 × 96	33 min and 20 s	0.343	0.080
551 × 96	37 min and 18 s	0.343	0.080

**Table 5:** Dimensionless temperature at X = L/2 at different nanofluid concentrations.

Volume Fraction	Dimensionless Temperature at X = L/2
4%	0.3408
8%	0.3425
10%	0.3433

**Table 6:** Average temperatures at the center of the cavity under different lid directions.

Direction	Positive		Negative	
Fluid	Dimensionless Temperature	Temperature (K)	Dimensionless temperature	Temperature (K)
Water	0.240	301.923	0.270	302.156
$SiO_{2}$ (Ri = 100)	0.245	302.558	0.276	302.888
$SiO_{2}$ (Ri = 77)	0.260	302.080	0.315	302.522

**Table 7:** Power generated under different cases.

Direction.	Power			
	Positive		Negative	
Fill factor	0.01	0.99	0.01	0.99
Water	2.240$\;{\times \;10}^{-3}$ μW/cm^2^	0.253 μW/cm^2^	2.270 ${\times \;10}^{-3}$ μW/cm^2^	0.318 μW/cm^2^
$SiO_{2}$ (Ri = 100)	4.04 ${\times \;10}^{-3}$μW/cm^2^	0.456 μW/cm^2^	5.047 ${\times \;10}^{-3}$ μW/cm^2^	0.569 μW/cm^2^
$SiO_{2}$ (Ri = 77)	2.630 ${\times \;10}^{-3}$ μW/cm^2^	0.297 μW/cm^2^	3.956 ${\times \;10}^{-3}$ μW/cm^2^	0.461 μW/cm^2^

**Table 8:** Power generated under different temperature gradients in the negative direction (ff = 0.99).

	ΔT			
	4 K	8 K	16	24
Water	0.245 K μW/cm^2^	0.435 K μW/cm^2^	0.919 K μW/cm^2^	1.43 K μW/cm^2^
$SiO_{2}$	0.195 K μW/cm^2^	0.31 K μW/cm^2^	0.68 K μW/cm^2^	1.01 K μW/cm^2^
Difference (%)	0.245 K μW/cm^2^	0.435 K μW/cm^2^	0.919 K μW/cm^2^	1.43 K μW/cm^2^

**Figure A1:**
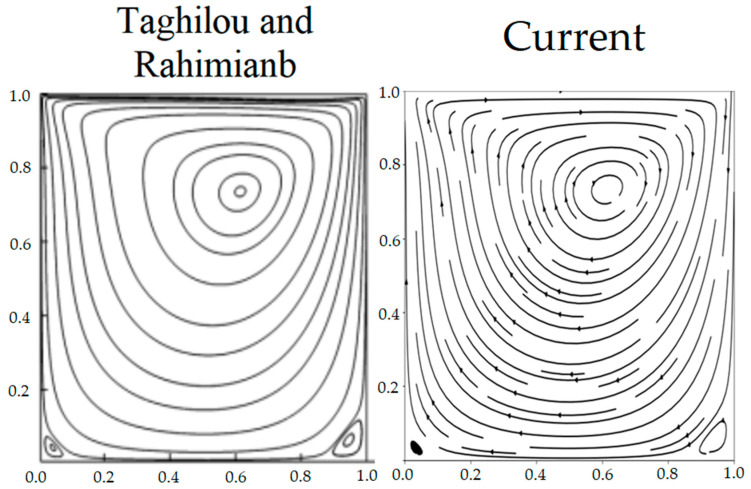
Vortex generated in a lid-driven cavity with Re = 100 Compared with Taghilou and Rahimianb [22].

**Figure A2:**
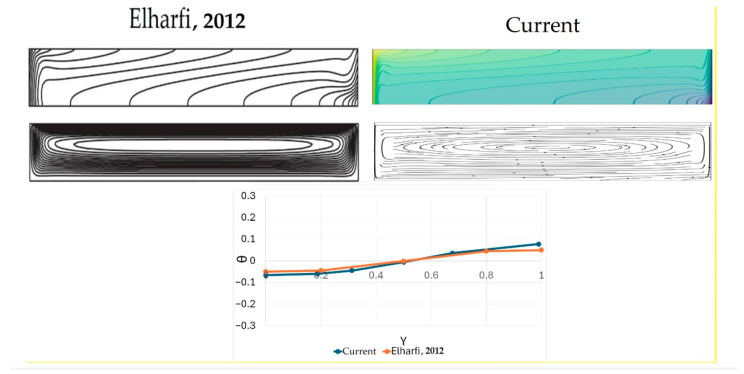
Streamlines, isotherms, and dimensionless temperature at Ri = 100 Compared with Elharfi et al. [6].

## Data Availability

The original contributions presented in this study are included in the article. Further inquiries can be directed to the corresponding author.

## Appendix A

Additional validations were made in a mesh of 401 × 96; the first presented in Figure A1 was made with Taghilou and Rahimianb [22], where flow in a lid-driven cavity with relation aspect of 1 was realized at a Reynolds number of 100. The images present similar behavior in the different vortices generated. Also, the center of the left vortex and the center of the right vortex presented a similar location: X = 0.0632 and Y = 0.9425 in the present work and X = 0.0625, Y = 0.9453 in previous investigations.

The second additional validation, presented in Figure A2, was performed by Elharfi et al. A simulation of nanofluid Cu–water with a volume fraction of 10% in a rectangular cavity with an aspect ratio of 8 was performed. The isotherms and streamlines present good similarity, and the dimensionless temperatures at X = L/2 present similar behavior.
